# Bcl-2 Family Members Bcl-xL and Bax Cooperatively Contribute to Bortezomib Resistance in Mantle Cell Lymphoma

**DOI:** 10.3390/ijms232214474

**Published:** 2022-11-21

**Authors:** Sudjit Luanpitpong, Montira Janan, Juthamas Yosudjai, Jirarat Poohadsuan, Pithi Chanvorachote, Surapol Issaragrisil

**Affiliations:** 1 Siriraj Center of Excellence for Stem Cell Research, Faculty of Medicine Siriraj Hospital, Mahidol University, Bangkok 10700, Thailand; 2 Department of Pharmacology and Physiology, Faculty of Pharmaceutical Sciences, Chulalongkorn University, Bangkok 10330, Thailand; 3 Cell-Based Drug and Health Product Development Research Unit, Faculty of Pharmaceutical Sciences, Chulalongkorn University, Bangkok 10330, Thailand; 4 Division of Hematology, Department of Medicine, Faculty of Medicine Siriraj Hospital, Mahidol University, Bangkok 10700, Thailand; 5 Bangkok Hematology Center, Wattanosoth Hospital, BDMS Center of Excellence for Cancer, Bangkok 10310, Thailand

**Keywords:** mantle cell lymphoma, bortezomib resistance, Bcl-2 family proteins, Bcl-xL, Bax, *O*-GlcNAcylation

## Abstract

Mantle cell lymphoma (MCL) is an aggressive non-Hodgkin lymphoma with poor prognosis, due to the inevitable development of drug resistance. Despite being the first-in-class proteasome inhibitor for relapsed/refractory MCL, resistance to bortezomib (BTZ) in MCL patients remains a major hurdle of effective therapy, and relapse following BTZ is frequent. Understanding the mechanisms underlying BTZ resistance is, therefore, important for improving the clinical outcome and developing novel therapeutic strategies. Here, we established de novo BTZ-resistant human MCL-derived cells with the highest resistance index of 300-fold compared to parental cells. We provided compelling evidence that both Bcl-xL and Bax are key mediators in determining BTZ sensitivity in MCL cells. Overexpression of antiapoptotic Bcl-xL and depletion of proapoptotic Bax cooperatively protected MCL cells against BTZ-induced apoptosis, causing acquired BTZ resistance, likely by tilting the balance of Bcl-2 family proteins toward antiapoptotic signaling. Bioinformatics analyses suggested that high *BCL2L1* (encoded Bcl-xL) and low *BAX* were, in part, associated with poor prognosis of MCL patients, e.g., when combined with low *OGT,* which regulates cellular *O*-GlcNAcylation. Our findings support recent strategies in small molecule drug discovery co-targeting antiapoptotic Bcl-2 family proteins using BH3 mimetics and Bax using Bax activators to overcome cancer drug resistance.

## 1. Introduction

Mantle cell lymphoma (MCL) is an aggressive non-Hodgkin lymphoma (NHL) arising from the pre-germinal center of a mature B cell that is characterized by the presence of translocation t(11;14)(q13;q32) resulting in an aberrant expression of cyclin D1 (encoded by *CCND1*) and uncontrolled cell growth [1,2]. The disease accounts for 3% to 6% of all NHL cases and is predominantly male, with a male-to-female ratio of approximately 3:1. Although some patients may follow an indolent clinical evolution, most have the clinical pattern of poor response to conventional chemotherapy and frequent relapse. MCL remains the worst prognosis among all NHL subtypes, with median overall survival (OS) of approximately 3–5 years following initial induction therapy and median OS of 1–2 years in patients with refractory or relapsed disease [3].

Bortezomib (BTZ) is a Food and Drug Administration (FDA)-approved first-in-class proteasome inhibitor for the treatment of relapsed/refractory MCL with an overall response rate (ORR) of 30–50%, irrespective of their sensitivity to prior therapy [4,5,6], suggesting little cross-resistance with conventional therapy. However, more than half of all MCL patients are either intrinsically resistant to BTZ or subsequently acquire resistance [7,8]. Drug resistance is a manifestation of cancer and is associated with somatic mutations and genomic plasticity. In this regard, numerous mechanisms associated with molecular changes of drug efflux pumps, drug metabolism, cell death inhibition, enhanced DNA repair, target gene amplification, and epigenetics have been shown to protect various cancers against chemotherapy [9,10,11]. Although the information on BTZ resistance in MCL remains largely unknown, attributable in part to its nature as a multifactorial phenomenon, certain characteristics of BTZ-resistant MCL cells have previously been reported, namely uncontrolled plasmacytic differentiation [12], activation of B-cell receptor (BCR) signaling [13], and alteration of lipid metabolism [14].

The aim of this study was to identify target candidates associated with BTZ resistance in MCL cells that could also predict the apoptosis response to BTZ. We established de novo BTZ-resistant MCL cells and profiled gene signatures involved in drug resistance and cell survival of MCL using sets of quantitative polymerase chain reaction (qPCR). We identified the involvement of Bcl-2 family members in BTZ response, stemming from the observed increase in antiapoptotic Bcl-xL (encoded by *BCL11A*) and a decrease in proapoptotic Bax (encoded by *BAX*) at both gene and protein levels in acquired BTZ-resistant MCL cells. We also performed bioinformatics database analyses to find an association of *BCL11A*, *BAX* and other reported genes involved in MCL aggressiveness with the clinical outcomes of MCL patients. Our findings provide better understanding of the mechanisms of BTZ resistance, which could be important in developing novel adjuvants for aggressive MCL.

## 2. Results

### 2.1. Establishment of BTZ-Resistant MCL Cells and Degrees of Resistance

**Figure 1:**
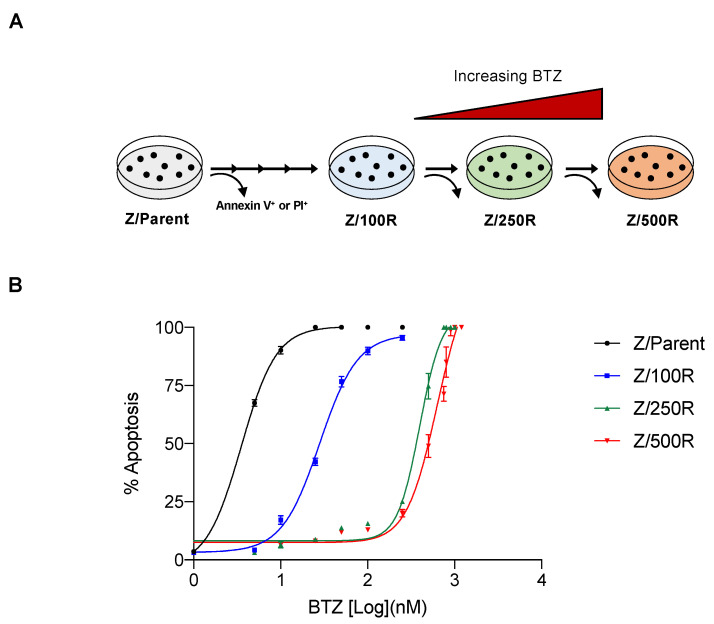
Generation of de novo BTZ-resistant MCL cells. (**A**) Schematic diagram for the establishment of BTZ-resistant Z-138 cells at different degrees from parental (Z/Parent) cells by stepwise selection method. (**B**) Parental Z/Parent cells and BTZ-resistant Z/100R, Z/250R, and Z/500R cells were treated with various concentrations of BTZ (0–1200 nM), and apoptosis was determined by the Hoechst 33342 assay at 24 h. Dose-response (best-fit) curves were generated on the logarithmic scale of drug concentrations as opposed to the linear scale of percentage of apoptosis. Data are mean ± SD (*n* = 3).

To investigate the molecular mechanisms of BTZ resistance in MCL, BTZ-resistant cells with acquired resistance were obtained from BTZ-sensitive human MCL-derived Z-138 (Z/Parent) cells after sequential treatments with increasing concentrations of BTZ, i.e., up to 500 nM. BTZ-resistant cells at different degrees of resistance were designated Z/100R, Z/250R, and Z/500R cells. Dose-response curves, which were generated from BTZ-induced apoptosis as evaluated by Hoechst 33342 assay at 24 h, displayed the sensitivity of BTZ-resistant cells compared to the parental Z/Parent cells (Figure 1A). The lethal concentration 50 (LC50), which caused half of the cells to undergo apoptosis, was calculated from the best-fit curves to the pooled data, and the reported values were 28.11 nM in Z/100R, 396.10 nM in Z/250R, and 628.3 nM in Z/500R cells versus 3.52 nM in Z/Parent cells. Resistance index (R) was then calculated from the relationships of LC50 of resistant cells to the LC50 of parental cells. The degrees of resistance in established resistant cells were approximately 8, 100, and 300-fold more resistant to BTZ than the parental cells.

**Figure 2:**
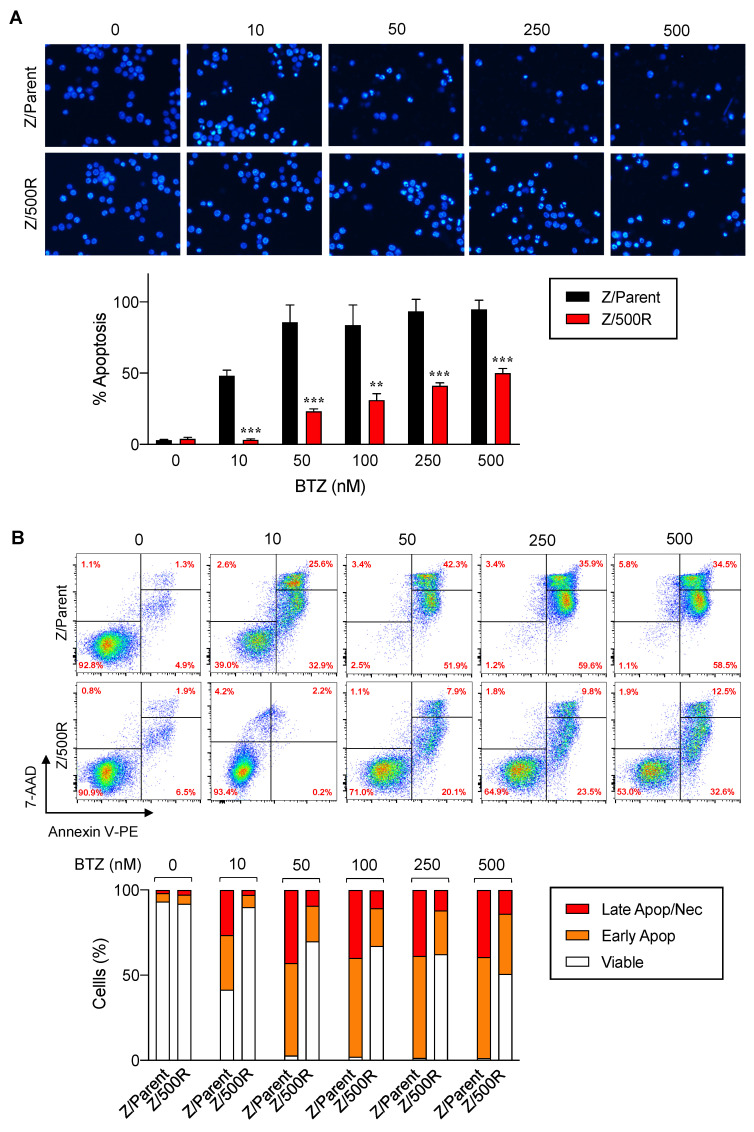
Resistant phenotype of the highly BTZ-resistant MCL cells in comparison to parental cells. (**A**) Parental Z/Parent and highly BTZ-resistant Z/500R cells were treated with BTZ (0–500 nM) and apoptosis was evaluated by the Hoechst 33342 assay at 24 h. (**Upper**) Representative micrographs of Hoechst 33342 nuclear staining under an inverted fluorescence microscope. (**Lower**) Percentages of apoptotic cells, which displayed condensed and/or fragmented nuclei, were scored from the micrographs and plotted. Data are mean ± SD (*n* = 3). ** *p* < 0.01, *** *p* < 0.001 versus Z/Parent cells; two-sided Student’s *t*-test. (**B**) Cells were similarly treated with BTZ (0–500 nM) and cell death was determined by Annexin V/7-AAD assay. Percentages of Annexin V single-positive cells defining early apoptosis, and Annexin V and 7-AAD double-positive cells defining late apoptosis/necrosis combined with 7-AAD defining necrosis were plotted.

Representative micrographs of Z/Parent cells under the blue fluorescence of Hoechst 33342 clearly displayed greater numbers of fragmented and/or condensed nuclei, the typical features of apoptosis, than those of Z/500R cells in all tested concentrations of BTZ (Figure 2A). By comparing the percentage of apoptosis scored from the micrographs, we confirmed that Z/500R cells were significantly more resistant than Z/Parent cells in response to BTZ at 10–500 nM. In agreement with the data obtained from Hoechst 33342 assay, Annexin V/7-AAD assays using flow cytometry validated that Z/500R cells were highly resistant to apoptosis induced by BTZ with a death rate of approximately 30% at 50 nM BTZ, the concentration that killed most, if not all, Z/Parent cells (Figure 2B). The death rate of Z/500R cells remained at approximately 50% even when treated with an extremely high concentration of BTZ at 500 nM. By comparing the percentage of total cell death and percentage of apoptosis of Z/Parent cells under the same conditions, we found that apoptosis was the mode of cell death induced by BTZ in these experimental settings.

### 2.2. Gene Expression Profiling of BTZ-Resistant MCL Cells and Pathway Analysis

**Figure 3:**
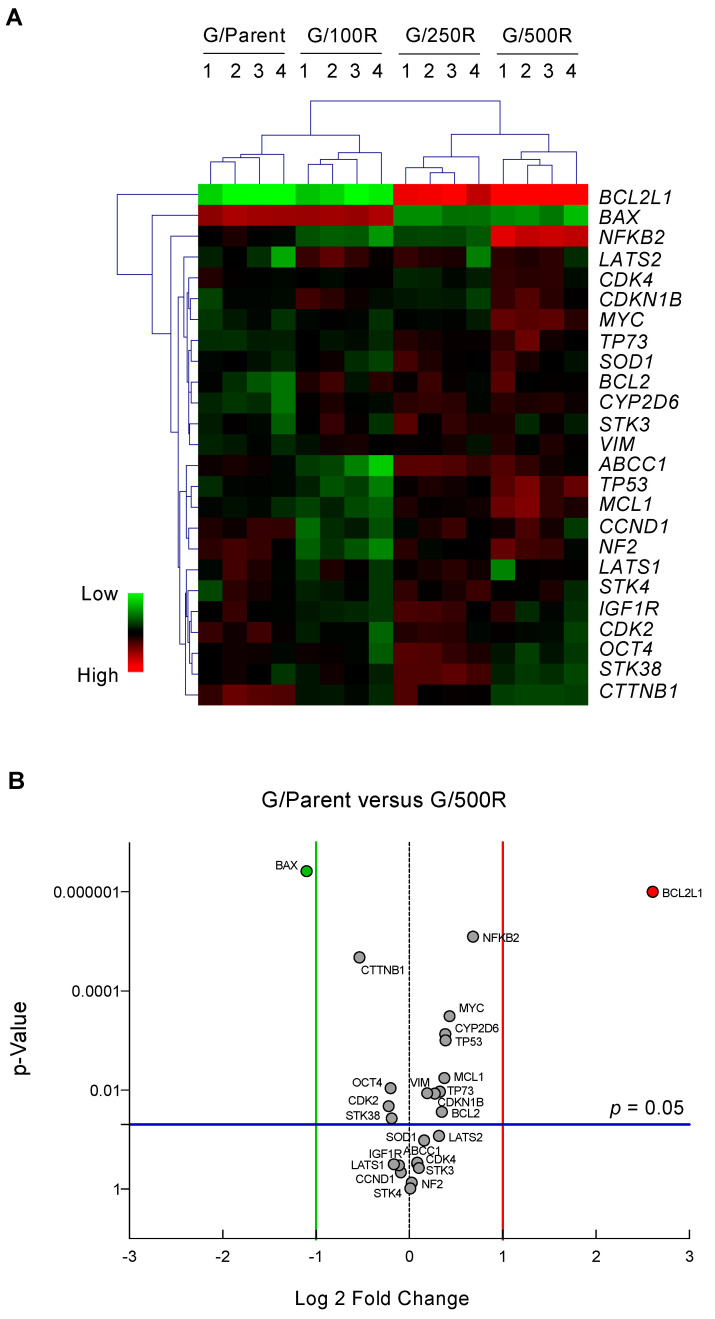
qPCR analysis of mRNA expression of putative cancer-related genes involved in drug resistance and cell survival of MCL. (**A**) Gene expression data in parental Z/Parent cells and BTZ-resistant Z/100R, Z/250R, and Z/500R cells from four independent experiments were normalized to housekeeping *GAPDH*, aligned, clustered, and represented in a heatmap. (**B**) Volcano plot of differentially expressed genes comparing Z/Parent and Z/500R cells with fold change > 2 and *p* < 0.05; two-sided Student’s *t*-test.

To identify genes and pathways important in the development of BTZ resistance, we profiled putative cancer-related genes involved in drug resistance and cell survival of MCL using sets of qPCR. These genes were grouped into: (i) drug efflux pumps *ABCB1*, *ABCC1*, and *ABCG2* [9]; (ii) drug metabolizing enzymes *CYP1A2*, *CYP2D6*, and *CYP3A4* [10]; (iii) apoptosis regulators *BCL2*, *BCL2L1*, *MCL1*, *BAX*, *TP53*, and *TP73* [11]; (iv) cell cycle regulators *CCND1*, *CDK2*, *CDK4*, and *CDKN2A* [15]; (v) cell survival and growth factors *NFKB2*, *MYC*, *SOD1*, *STK38*, and *IGF1R* [16,17,18]; (vi) Hippo pathway components *NF2*, *LATS1*, *LATS2*, *STK3*, and *STK4* [19], and (vii) clonal regulators *VIM* and *OCT4* [20]. Gene expression levels normalized to the housekeeping *GAPDH* comparing parental Z/Parent cells and BTZ-resistant Z/100R, Z/250R and Z/500R cells were aligned, clustered, and represented in a heatmap. Notably, *ABCB1*, *ABCG2*, *CYP1A2*, and *CYP3A4* were expressed at barely detectable levels in both parental and BTZ-resistant cells, hence they were excluded from the analyses. Figure 3A shows that *BCL2L1* and *BAX* are the top-ranked genes that clearly distinguish Z/Parent and Z/100R cells from the highly resistant Z/250R and Z/500R cells. Next, a volcano plot was created to better visualize the differential gene expression in Z/Parent versus Z/500R cells using *p* < 0.05 and ± two-fold change filter criteria. Figure 3B further confirms that only Bcl-2 family members *BCL2L1* and *BAX* passed the criteria—*BCL2L1* was significantly upregulated, while *BAX* was conversely downregulated in Z/500R cells when compared to Z/Parent cells. Therefore, *BCL2L1* and *BAX* were chosen as target candidates in the present study.

**Figure 4:**
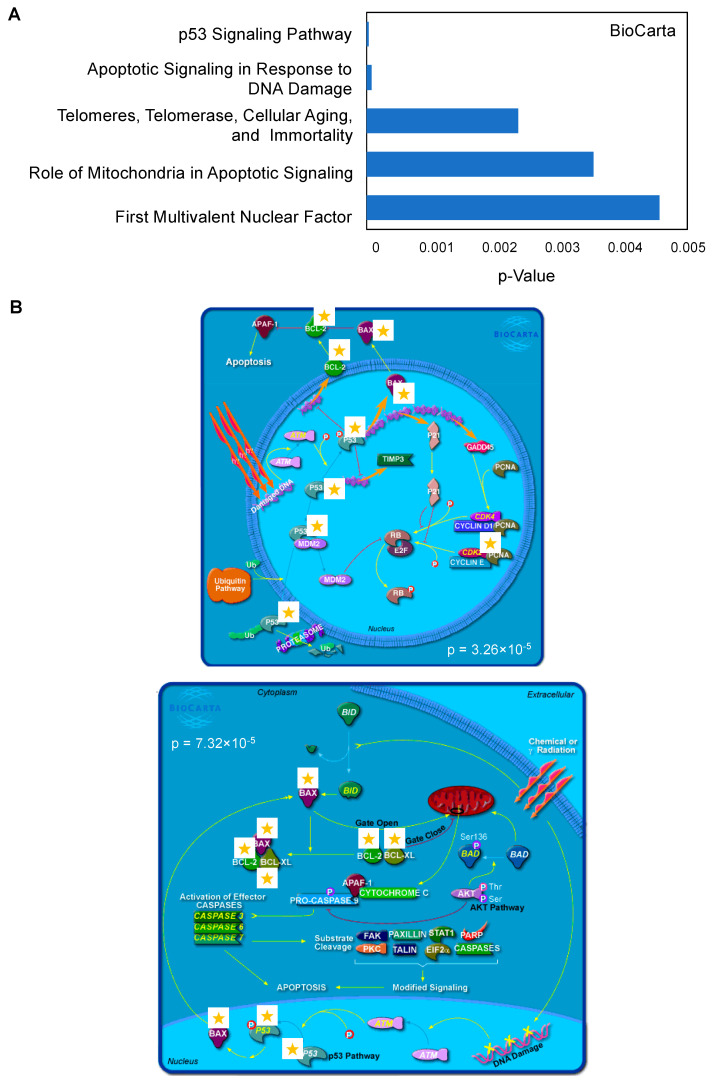
Top-ranked pathways associated with BTZ resistance in MCL cells. (**A**) Lists of top-ranked BioCarta pathways generated from lists of significantly changed genes between parental Z/Parent and BTZ-resistant Z/500R cells. (**B**) Schematic diagrams of the p53 signaling pathway (**upper**) and apoptosis signaling in response to DNA damage (**lower**) generated by BioCarta. Yellow stars indicate listed genes.

To gain better insight into the pathways associated with BTZ resistance, we input the significantly changed genes between Z/Parental and Z/500R cells (*p* < 0.05) into the database for Annotation, Visualization and Integrated Discovery (DAVID) functional annotation tool, and BioCarta pathway analysis was performed [21,22]. Figure 4A shows a list of top-ranked pathways, including the p53 signaling pathway and apoptosis signaling, in response to DNA damage, which are schematically illustrated in Figure 4B. The limitation of the analyses was the use of a predefined gene list in which only those genes known to be involved in drug resistance and cell survival were included. It is worth noting that our studies do not exclude the involvement of additional signaling pathways. This requires further investigation.

### 2.3. BTZ-Resistant MCL Cells Displayed Elevated Bcl-xL and Repressed Bax Levels

**Figure 5:**
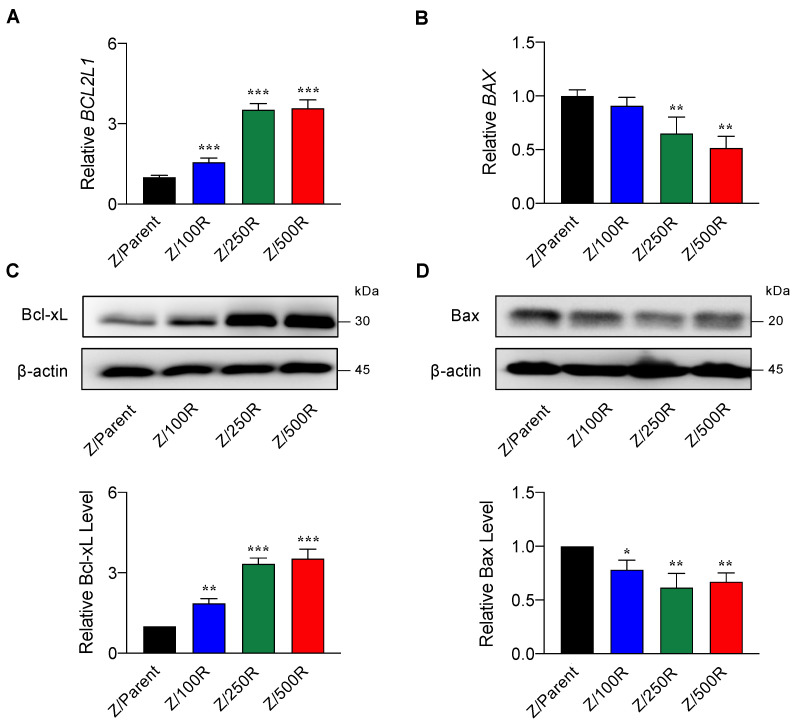
Determination of Bcl-2 and Bax levels in BTZ-resistant cells in comparison to their gene expression. (**A**,**B**) mRNA expression of *BCL2L1* (encodes Bcl-xL) and *BAX* in parental Z/Parent cells and BTZ-resistant Z/100R, Z/250R, and Z/500R cells using qPCR. Data are mean ± SD (*n* = 3). ** *p* < 0.01, *** *p* < 0.001 versus Z/Parent cells; two-sided Student’s *t*-test. (**C**,**D**) Western blot analysis of Bcl-xL and Bax levels in Z/Parent, Z/100R, Z/250R, and Z/500R cells. Data are mean ± SD (*n* = 3). * *p* < 0.05, ** *p* < 0.01, *** *p* < 0.001 versus Z/Parent cells; two-sided Student’s *t*-test.

Having demonstrated that *BCL2L1* and *BAX* were differentially expressed in the highly resistant Z/500R cells when compared to Z/Parent cells (Figure 3B), we next evaluated whether Bcl-xL and Bax protein levels correlated well with the degree of BTZ resistance. Figure 5B shows a remarkable increase in Bcl-xL level when the cells acquired higher resistance, which was correlated well with its gene expression (Figure 5A), suggesting that the regulation of Bcl-xL in BTZ-resistant cells occurs mainly at the transcriptional level. By contrast, Bax level decreased by approximately 30–40% in all BTZ-resistant cells regardless of its gene expression and degree of resistance, suggesting that post-translational modifications might be involved in the regulation of Bax in BTZ-resistant cells.

### 2.4. Overexpression of Bcl-xL Contributes to Acquired BTZ Resistance

**Figure 6:**
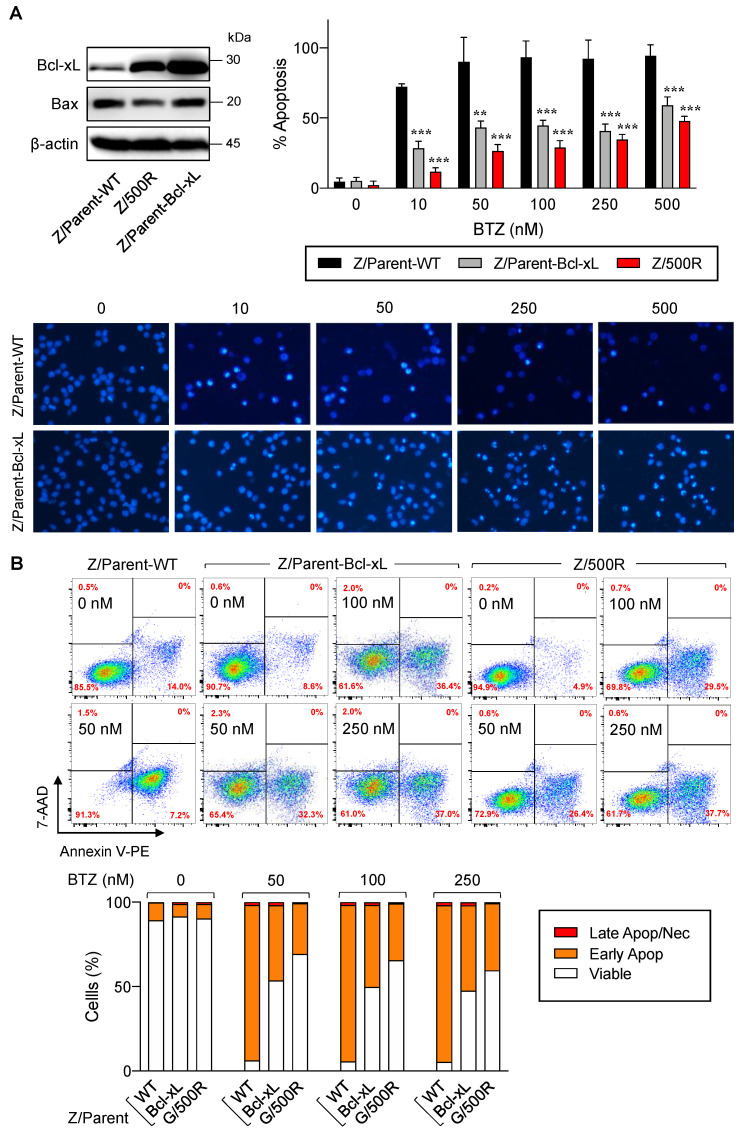
Bcl-xL is a key mediator of BTZ-induced apoptosis. Parental Z/Parent cells were transduced with Bcl-xL retroviral particles or control particles (WT) and treated with BTZ (0–500 nM) for 24 h. (**A**) **Upper left**: Western blot analysis of Bcl-xL level, along with Bax level, in Z/Parent-WT and Z/Parent-Bcl-xL cells in comparison to the highly BTZ-resistant Z/500R cells prior to experiments. **Upper**, **right**: Percentages of apoptosis as evaluated by Hoechst 33342 assay were scored and plotted. Data are mean ± SD (*n* = 3). ** *p* < 0.01, *** *p* < 0.001 versus Z/Parent-WT cells; two-sided Student’s *t*-test. **Lower**: Representative micrographs of Hoechst 33342 nuclear staining under an inverted fluorescence microscope. (**B**) Analysis of cell death by Annexin V/7-AAD assay. Percentages of Annexin V single-positive cells defining early apoptosis, and Annexin V and 7-AAD double-positive cells defining late apoptosis/necrosis combining with 7-AAD single-positive cells defining necrosis were plotted.

The Bcl-2 family is the best characterized family of proteins involved in the regulation of apoptosis, consisting of antiapoptotic and proapoptotic members. The main function of antiapoptotic Bcl-2 family proteins is to restrain proapoptotic Bcl-2 family proteins primarily by direct binding interactions, thus preserving mitochondrial outer membrane permeabilization (MOMP) and preventing the subsequent release of cytochrome C [23,24]. To first evaluate whether antiapoptotic Bcl-xL rendered MCL cells apoptosis-resistant to BTZ, we introduced Bcl-xL transgene into parental Z/Parent cells using retroviral particles, hereafter designated as Z/Parent-Bcl-xL cells, and evaluated their apoptosis in response to BTZ in comparison to their counterpart control Z/Parent cells and the highly resistant Z/500R cells. All cells were treated with BTZ (0–500 nM), and apoptosis was evaluated by Hoechst 33342 and Annexin V/7-AAD assays at 24 h. Western blot analysis was performed to ascertain an increase in Bcl-xL level in Z/Parent-Bcl-xL cells (Figure 6A). Bax level was also checked and was found to be minimally changed in Z/Parent-Bcl-xL cells. By nuclear staining, the percentage of apoptosis was significantly lower in Z/Parent-Bcl-xL and Z/500R cells when compared to Z/Parent cells. Representative micrographs that clearly exhibit the lesser extent of cells with condensed and/or fragmented nuclei in Z/Parent-Bcl-xL cells when compared to Z/Parent cells are shown in the lower panel. Further, the results from Annexin V/7-AAD assay validated that Bcl-xL overexpression protected the Z/Parent cells from cell death induced by BTZ (10–500 nM) (Figure 6B). Notably, we observed that overexpression of Bcl-xL alone could cause the Z/Parent cells to acquire the BTZ-resistant phenotype to a degree that was almost comparable to those of Z/500R cells, although we did not make a direct comparison considering other confounding variables. Altogether, these results indicate the important role of Bcl-xL in BTZ sensitivity of MCL cells.

### 2.5. Depletion of Bax Similarly Leads to BTZ Resistance

**Figure 7:**
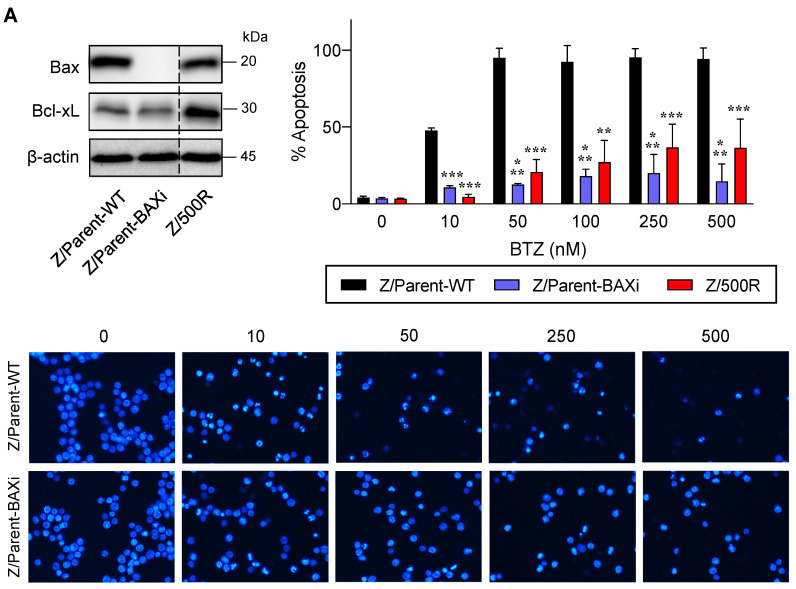

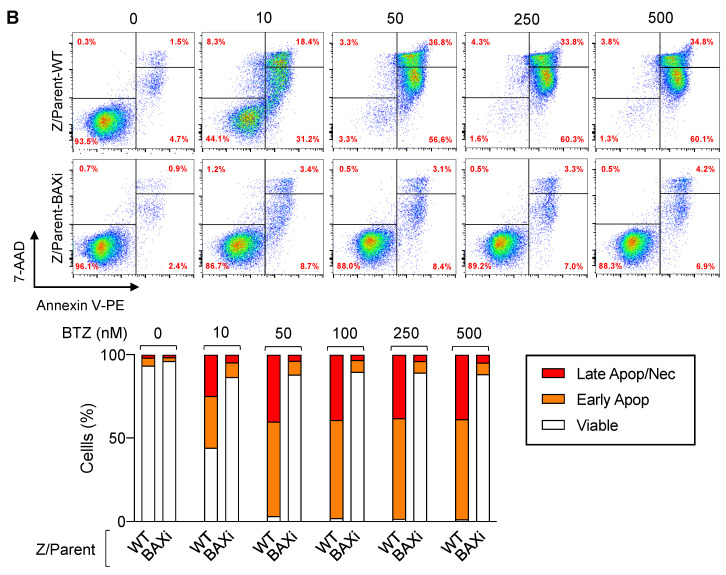
Bax is also a key mediator of BTZ-induced apoptosis. Parental Z/Parent cells were transduced with lentiviral particles carrying Cas9 and sgRNA against human *BAX* or control particles (WT) and treated with BTZ (0–500 nM) for 24 h. (**A**) **Upper left**: Western blot analysis of Bax level, along with Bcl-xL level, in Z/Parent-WT and Z/Parent-BAXi cells in comparison to the highly BTZ-resistant Z/500R cells prior to experiments. The dashed line separates juxtaposed lanes taken from the same experiment and blot image. **Upper right**: Percentages of apoptosis as evaluated by Hoechst 33342 assay were scored and plotted. Data are mean ± SD (*n* = 3). ** *p* < 0.01, *** *p* < 0.001 versus Z/Parent-WT cells; two-sided Student’s *t*-test. **Lower**: Representative micrographs of Hoechst 33342 nuclear staining under an inverted fluorescence microscope. (**B**) Analysis of cell death by Annexin V/7-AAD assay. Percentages of Annexin V single-positive cells defining early apoptosis, and Annexin V and 7-AAD double-positive cells defining late apoptosis/necrosis combined with 7-AAD single-positive cells defining necrosis were plotted.

Bax is a proapoptotic Bcl-2 family protein that, together with its homolog protein Bak, is considered a core regulator of the intrinsic pathway of apoptosis [24,25]. Bax and Bak, which form pores in the mitochondrial outer membrane upon their conversion from harmless monomers into deadly oligomers, are regulated by various antiapoptotic Bcl-2 family proteins, including Bcl-xL. To identify the functional role of Bax in BTZ response in MCL cells, the CRISPR/Cas9 system was used to deplete Bax in the parental Z/Parent cells, and their effect on apoptosis was evaluated using Hoechst 33342 and Annexin V/7-AAD assays. Similar to the overexpression of Bcl-xL (Figure 6), depletion of Bax conferred BTZ resistance to Z/Parent cells to a degree that resembled the highly resistant Z/500R cells (Figure 7A,B), indicating that loss of Bax efficiently protected the Z/Parent cells from apoptosis by BTZ (10–500 nM) and that acquired BTZ resistance was regulated in part by Bax, although its protein level was not correlated well with the degree of resistance in BTZ-resistant cells.

### 2.6. Overexpression of Bcl-xL and Depletion of Bax Cooperatively Caused More BTZ-Resistant MCL Cells

**Figure 8:**
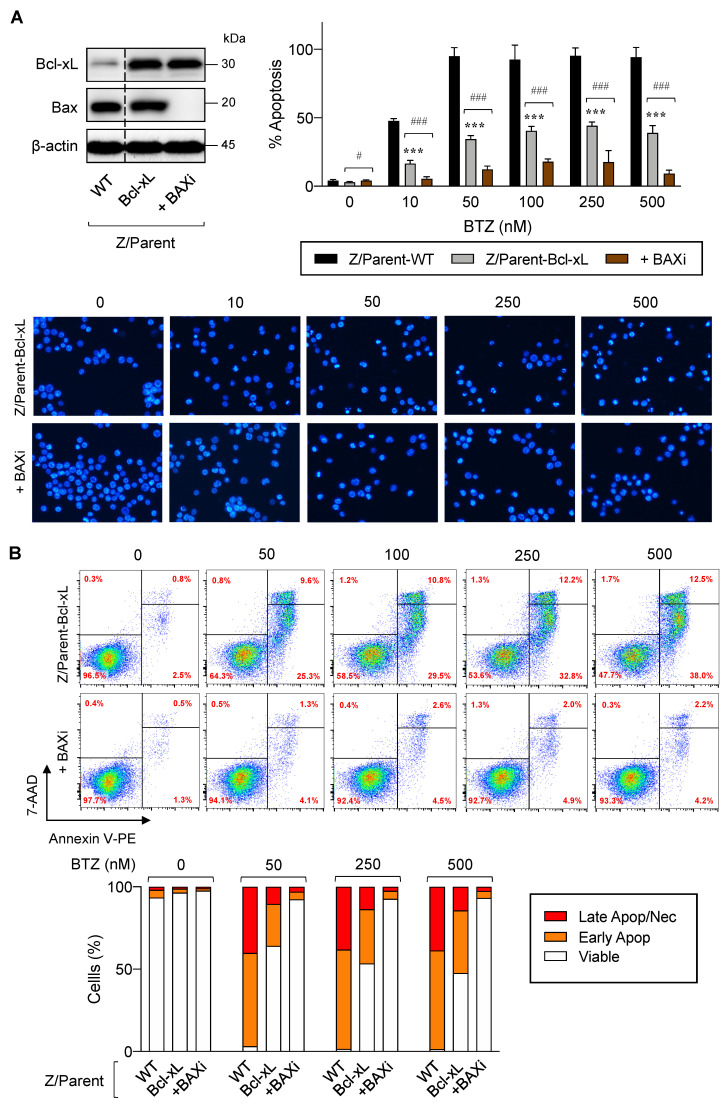
Overexpression of Bcl-xL and depletion of Bax cooperatively induces acquired BTZ resistance in MCL cells. Bcl-xL-overexpressed Z-138 cells were transduced with lentiviral particles carrying Cas9 and sgRNA against human *BAX* (Z/Parent-Bcl-XL + BAXi) or control particles (Z/Parent-Bcl-xL) and treated with BTZ (0–500 nM) for 24 h. (**A**) **Upper left**: Western blot analysis of Bcl-xL and Bax levels in Z/Parent-Bcl-xL + BAXi and Z/Parent-Bcl-xL cells in comparison to the parental Z/Parent-WT cells prior to experiments. The dashed line separates juxtaposed lanes taken from the same experiment and blot image. **Upper right**: Percentages of apoptosis as evaluated by Hoechst 33342 assay were scored and plotted. Data are mean ± SD (*n* = 3). *** *p* < 0.001 versus Z/Parent-WT cells; ^#^ *p* < 0.05, ^###^ *p* < 0.001 versus Z/Parent-Bcl-xL cells; two-sided Student’s *t*-test. **Lower**: Representative micrographs of Hoechst 33342 nuclear staining under an inverted fluorescence microscope. (**B**) Analysis of cell death by Annexin V/7-AAD assay. Percentages of Annexin V single-positive cells defining early apoptosis, and Annexin V and 7-AAD double-positive cells defining late apoptosis/necrosis combining with 7-AAD single-positive cells defining necrosis were plotted.

Previous studies have reported that cancer cells commonly evade apoptosis by upregulation of the antiapoptotic Bcl-2 family proteins. In more resistant cells, downregulation or inactivation of proapoptotic Bcl-2 family proteins, particularly BH3-only proteins, also occurs to suppress apoptosis [26,27]. To test whether depletion of Bax, the proapoptotic pore-former, directly synergized with the protective effect of Bcl-xL on BTZ-induced apoptosis, Bax was inhibited in Z/Parent-Bcl-xL cells using the CRISPR/Cas9 system. Figure 8A,B shows that Z/Parent-Bcl-xL + BAXi cells were significantly less responsive to apoptosis in response to BTZ (10–500 nM) when compared to the counterpart control Z/Parent-Bcl-xL cells, indicating that Bax worked in concert with Bcl-xL in evading BTZ-induced apoptosis and acquiring BTZ resistance.

**Figure 9:**
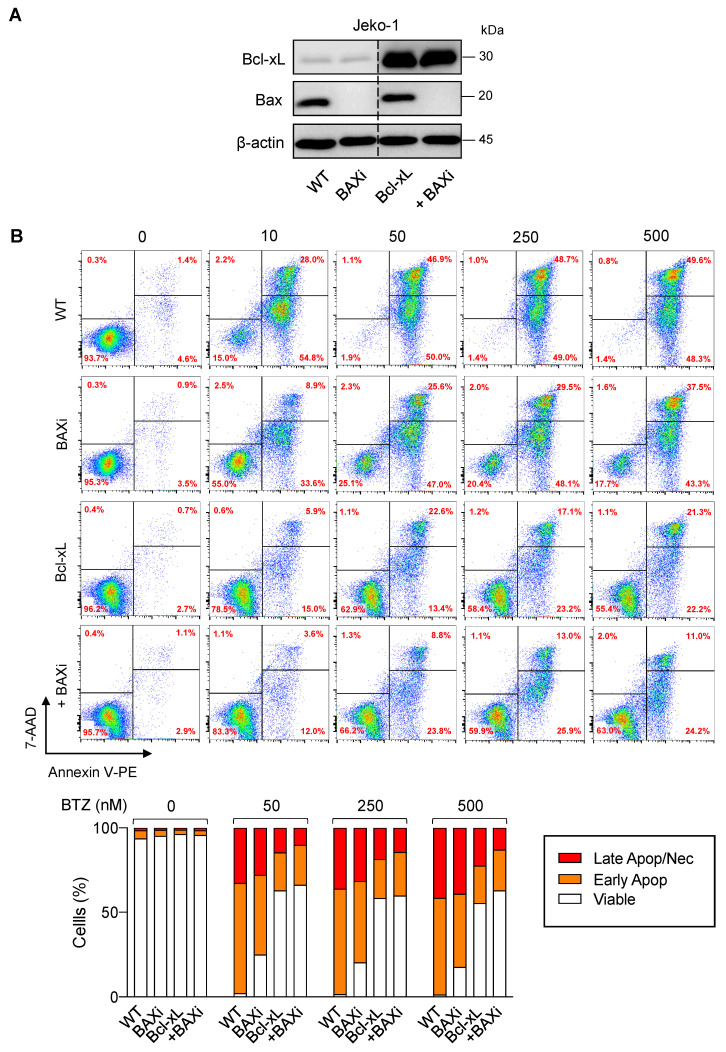
Generality of the observed Bcl-xL and Bax effects in BTZ-induced apoptosis in MCL cells. Genetic manipulation of Bcl-xL and Bax was similarly performed in human MCL-derived Jeko-1 cells using transgene and/or CRISPR/Cas9 system. After which, cells were treated with BTZ (0–500 nM) for 24 h. (**A**) Western blot analysis of Bcl-xL and Bax levels in control Jeko-1 (WT), Bax-depleted (BAXi), Bcl-xL-overexpressed alone (Bcl-xL) or in combination with Bax depletion (Bcl-xL + BAXi) cells prior to experiments. The dashed line separates juxtaposed lanes taken from the same experiment and blot image. (**B**) Analysis of cell death by Annexin V/7-AAD assay. Percentages of Annexin V single-positive cells defining early apoptosis, and Annexin V and 7-AAD double-positive cells defining late apoptosis/necrosis combining with 7-AAD single-positive cells defining necrosis were plotted.

To substantiate the findings on the functional role of Bcl-xL and Bax in determining BTZ sensitivity in MCL cells, BTZ-sensitive human MCL-derived Jeko-1 cells were used, and genetic manipulation of Bcl-xL and Bax alone or in combination, was performed as previously described in Z-138 cells. Western blot analysis was performed to ascertain the successful overexpression of Bcl-xL and/or depletion of Bax (Figure 9A) prior to treatment with BTZ (10–500 nM). Cell death was then determined by the Annexin V/7-AAD assay at 24 h. The results showed that overexpression of Bcl-xL and, to a much lesser extent, depletion of Bax alone, could render Jeko-1 cells resistant to apoptosis induced by BTZ (10–500 nM), suggesting the generality of their roles in BTZ-induced apoptosis in MCL cells (Figure 9B). We, however, noticed that depletion of Bax in Bcl-xL-overexpressed cells minimally improved BTZ response when compared to their counterpart control Bcl-xL cells, which is likely due to the already very high protection of Bcl-xL alone against BTZ-induced apoptosis in Jeko-1 cells.

### 2.7. High BCL2L1 and Low BAX Are in Part Associated with Poor Prognosis

**Figure 10:**
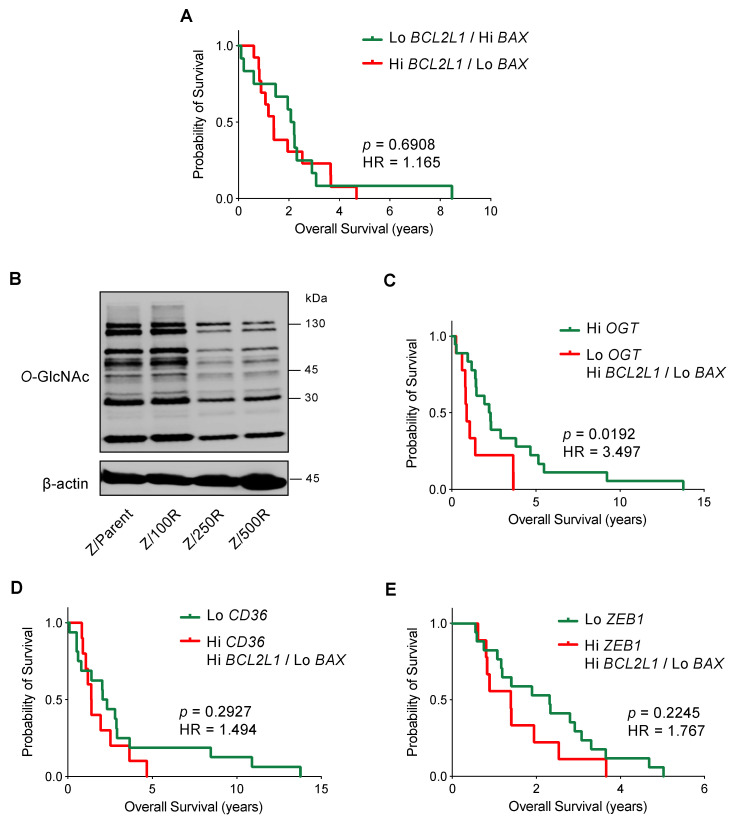
Kaplan-Meier survival plot of MCL patients from the gene microarrays (GSE10793) according to the levels of *BCL2L1*, *BAX*, *OGT*, *CD36*, and *ZEB1* expression. Values above the upper quartile (Q3) were defined as high expression. (**A**) Overall survival of patients with high *BCL2L1* and low *BAX* (red line) is compared to patients with low *BCL2L1* and high *BAX* (green line). (**B**) Western blot analysis of global *O*-GlcNAc level in parental Z/Parent and BTZ-resistant Z/100R, Z/250R, and Z/500R cells showing a dose-dependent decrease in cellular *O*-GlcNAcylation when the cells acquired higher BTZ resistance. (**C**) Overall survival of patients with low *OGT* together with high *BCL2L1* and low *BAX* (red line) is compared to patients with high *OGT* (green line). (**D**) Overall survival of patients with high *CD36* together with high *BCL2L1* and low *BAX* (red line) is compared to patients with low *CD36* (green line). (**E**) Overall survival of patients with high *ZEB1* together with high *BCL2L1* and low *BAX* (red line) is compared to patients with low *ZEB1* (green line). HR, hazard ratio.

Having shown that Bcl-xL activation and/or Bax depletion dictates the BTZ-resistant phenotype in MCL cells, this section outlines how Bcl-xL and Bax might correlate with the clinical outcome. Since it is well-accepted that drug resistance is dependent on multiple mechanisms, we performed mRNA expression analysis of *BCL2L1*, *BAX*, and other genes reported to be important regulators of BTZ resistance in MCL cells, including *OGT*, *CD36*, and *ZEB1,* in human MCL microarrays available on Gene Expression Omnibus (GEO; accession number GSE10793) [28] to examine the association between their expression and survival of MCL patients. We first grouped patients according to their Bcl-2 family gene co-expression into patients with high *BCL2L1* (cutoff: above Q3) and low *BAX* (cutoff: below Q3), and patients with the opposite gene co-expression of low *BCL2L1* and high *BAX*. After which, an additional gene of interest was added to the co-expression analysis. Figure 10A shows a similar outcome between the two groups of Bcl-2 family gene co-expression.

*O*-GlcNAcylation is an essential posttranslational modification and an ideal metabolic sensor in the hexosamine biosynthetic pathway that is regulated by two cycling enzymes *O,*-GlcNAc transferase (OGT; encoded by *OGT*) and *O*-GlcNAcase (OGA encoded by *MGEA5*) [29,30]. We recently reported that an increase in cellular *O*-GlcNAcylation, which could be endorsed by an inhibition OGA or alternatively an activation of OGT, sensitizes MCL cells to BTZ-induced apoptosis and reverses their apoptosis resistance via the stabilization of truncated Bid (tBid) [31]. Herein, we showed that BTZ-resistant Z-138 cells exhibited a decrease in cellular *O*-GlcNAcylation when compared to the parental cells, in association with the degree of BTZ resistance (Figure 10B). Our survival analysis revealed that MCL patients with low *OGT* (cutoff: below Q3) together with high *BCL2L1* and low *BAX* have shorter survival than those patients with high *OGT*: the hazard ratio increased by approximately three-fold. As the overall survival in patients with high *OGT* versus low *OGT* alone was not significantly different (*p* = 0.1700), our data suggest that high Bcl-xL and low Bax could be cooperative factors that. with decreased *O*-GlcNAcylation, are involved in controlling aggressive MCL (Figure 10C).

Dysregulated lipid metabolism in MCL cells via CD36 has been shown to be linked to BTZ resistance [14]. Likewise, Zeb-1, which was highly expressed in a cancer stem cell (CSC) subpopulation of MCL cells [32], induced lipid accumulation and correlated with BTZ resistance in MCL cells [14]. Figure 10D,E, however, shows that the overall survival between patients with high *CD36* or *ZEB1* (cutoff: above Q3) together with high *BCL2L1* and low *BAX,* and patients with low *CD36* or *ZEB1* (cutoff: below Q3) alone, was not significantly different, suggesting that Bcl-2 family proteins and CD36 or Zeb-1 might not cause a synergistic effect.

## 3. Discussion

MCL is a particularly deadly subtype of NHL with poor prognosis, attributable to its innate and acquired resistance to current chemotherapy regimens. Despite being the first-in-class proteasome inhibitor for relapsed/refractory MCL, resistance to BTZ in MCL patients remains a major hurdle of effective therapy, and relapse following BTZ is frequent [7,8,33]. Therefore, a better understanding of the mechanisms underlying BTZ resistance is crucial for developing novel strategies for better outcomes. Bcl-2 family proteins have been shown to be important in regulating apoptosis, including anoikis, during tumor progression and metastasis, and in regulating cellular responses to various chemotherapeutic drugs [34,35,36]. Activation of antiapoptotic Bcl-2 family proteins, such as Bcl-2, Mcl-1, and Bcl-xL, are frequently observed in chemoresistant tumor cells, offsetting the functions of proapoptotic Bcl-2 family proteins and suppressing apoptosis [37,38]. In MCL cells, dysregulation of Bcl-2 family proteins, including *BCL2* overexpression and *BIM* repression caused by genetic abnormalities, and *BCL2L1* upregulation caused by microenvironmental interactions, are detected [39,40]. Accordingly, antiapoptotic Bcl-2 family proteins have become important therapeutic targets for which several ongoing clinical trials have been conducted with Bcl-2 inhibitors, either alone or in combination with existing chemotherapeutic drugs.

Upon screening of several genes involved in drug resistance and cell survival of MCL, we found the striking upregulation of *BCL2L1* (encodes Bcl-xL) and downregulation of *BAX* in highly BTZ-resistant cells (Figure 3). Subsequent Western blot analyses suggested that the elevated Bcl-xL level in BTZ-resistant cells, which correlates well with its gene expression and the degree of BTZ resistance, is likely mediated via transcriptional program, while Bax level is additionally mediated at the posttranscriptional level (Figure 5). Reported PTMs of Bax include the phosphorylation at S184 and T167, which inactivates Bax, the dephosphorylation at T172, T174, and T176, which downregulates Bax, and the polyubiquitination at a non-specific site, which targets Bax for proteasomal degradation [24].

Overexpression of Bcl-xL has been reported in the majority of various hematologic malignancies, including Burkitt’s lymphoma (BL), diffuse large B cell lymphoma (DLBCL), acute myeloid leukemia (AML), acute lymphoblastic leukemia (ALL), and multiple myeloma (MM) [41,42]. Although little is known about the role of Bcl-xL in BTZ response in hematologic malignancies, a gradual loss of Bcl-xL, in concert with Noxa upregulation and its increased binding to Bcl-xL, was shown to be necessary for BTZ-induced apoptosis in neuroblastoma cells [43]. In murine hematopoietic cells, expression of bcl-xL caused multidrug resistance to chemotherapeutic agents, including bleomycin, cisplatin, vincristine, and etoposide [44]. Additionally, Bcl-xL has been linked to the acquired resistance of chronic lymphocytic leukemia (CLL) cells to ABT-199 (venetoclax), an orally bioavailable and selective Bcl-2 inhibitor approved for both treatment-naïve and relapsed CLL [45]. We herein report that overexpression of Bcl-xL desensitized various BTZ-sensitive MCL cells to BTZ-induced apoptosis (Figure 6 and Figure 9), highlighting the contribution of Bcl-xL to BTZ sensitivity.

Activation of the proapoptotic pore-formers Bax and Bak is an indispensable gateway to MOMP and consequently apoptosis. Downregulation and loss-of-function mutation of *BAX* were found to be associated with chemoresistance in various cancers [46]. A missense G179E *BAX* mutation, which abrogates Bax anchoring to mitochondria and blocks apoptosis, was found in ABT-199-resistant MCL cells. Interestingly, G179E *BAX* mutation could possibly explain the partial cross-resistance of ABT-199-resistant MCL cells to other cytotoxic drugs, including taxol, cisplatin, doxorubicin, temsirolimus, TRAIL, and BTZ [47]. In the present study, depletion of Bax in BTZ-sensitive MCL cells exerted protective effects against BTZ-induced apoptosis (Figure 7 and Figure 9). We earlier showed that BTZ treatment in MCL cells caused the induction of tBid [31], which could somehow recruit inactive Bax from the cytosol and induce its oligomerization and MOMP [24]. Thereby, depletion of Bax might interfere with the function of tBid in mediating BTZ-induced apoptosis.

The relative ratios of pro- and antiapoptotic Bcl-2 family proteins have been shown to determine the sensitivity of cells to various apoptotic stimuli, including loss of cell anchorage, growth factor deprivation, radiation, and chemotherapeutic drugs [48]. We showed that overexpression of Bax, together with depletion of Bcl-xL, mimicking the highly BTZ-resistant MCL cells, strongly tilted the balance toward antiapoptotic signaling and resulted in even higher protection against BTZ when compared to overexpression of Bcl-xL alone (Figure 8 and Figure 9). Notably, the cooperative effect of Bcl-xL and Bax was more noticeable in Z-138 cells, likely due to the existing strong protective effect of Bcl-xL alone on BTZ-induced apoptosis in Jeko-1 cells. A recent study showed that co-targeting of Bcl-xL and Bax by ABT-263 (navitoclax), an orally bioavailable inhibitor of Bcl-xL, Bcl-2, and Bcl-w, and BTSA1.2, an orally bioavailable Bax activator, synergistically increased cytotoxicity in many hematologic and solid tumor cell lines, regardless of common genetic alterations, such as *TP53* and *RAS* mutation, when compared to ABT-263 alone. Altogether, these findings strengthen the concept that co-targeting Bcl-xL and Bax is an attractive therapeutic strategy for overcoming cancer drug resistance [26]. Earlier, the idea of co-targeting Mcl-1 and Noxa in MCL cells was proposed by combining CDK inhibitor dinaciclib and fatty acid synthase (FASN) inhibitors, e.g., orlistat [49]. The cytotoxicity of dinaciclib, which caused Mcl-1 reduction, can be augmented by forced stabilization of Noxa using FASN inhibitors, thereby supporting that the balance of pro- and antiapoptotic Bcl-2 family proteins is critical in determining MCL cell fate.

BTZ resistance in MCL cells is a multifactorial phenomenon involving the CSC subpopulation, plasmacytic differentiation, and cellular metabolism [12,14,32]. Our bioinformatics analyses revealed that MCL patients with low *OGT* together with high *BCL2L1* and low *BAX* have poorer prognosis than those patients with high *OGT* (Figure 10). As cellular *O*-GlcNAcylation, which can be endored by either OGA inhibition or OGT activation, can enhance BTZ sensitivity in de novo BTZ-resistant cells via tBid [31], it is interesting to know that expression of *OGT*, *BCL2L1*, and *BAX* cooperatively predicts the clinical outcome in MCL patients.

## 4. Materials and Methods

### 4.1. Reagents

Bortezomib was obtained from Janssen-Cilag (Beerse, Belgium). Hoechst 33342 was obtained from Molecular Probes (Eugene, OR, USA). PE Annexin V Apoptosis Detection Kit I was obtained from BD Bioscience (BD Bioscience, San Jose, CA, USA). Antibodies for Bcl-xL, Bax, and β-actin were obtained from Cell Signaling Technology (Danvers, MA, USA), while an antibody for O-GlcNAc (RL2) was from Abcam (Cambridge, UK).

### 4.2. Cell Culture

Human MCL-derived Jeko-1 cells (RRID:CVCL_1865) were obtained from American Type Culture Collection (ATCC; Manassas, MA, USA), while human MCL-derived Z-138 cells (RRID:CVCL_B077) were a kind gift from Dr. Siwanon Jirawatnotai (Systems Pharmacology, Faculty of Medicine Siriraj Hospital, Bangkok, Thailand). STR analysis of the cells (16 loci) was performed at the Department of Forensic Medicine, Faculty of Medicine Siriraj Hospital, Mahidol University and the profiles can be found in Supplementary Tables S1 and S2. Cells were cultured in RPMI1640 medium supplemented with 10% fetal bovine serum (FBS), 2 mM L-glutamine, 100 U/mL penicillin and 100 µg/mL streptomycin at 37 °C and 5% CO_2_. Mycoplasma contamination was regularly checked using a commercial test kit (MycoAlert™ PLUS, Lonza, Cologne, Germany) and any cell lines found positive were discarded.

### 4.3. Generation of De Novo BTZ-Resistant Cells

BTZ-resistant Z-138 cells were generated by a stepwise selection method as described [14]. Parental MCL-derived Z-138 (Z/Parent) cells were continuously exposed to increasing concentrations of BTZ to the maximum concentration of 100, 250 and 500 nM. Resistant cells were examined by Annexin V-PE/7-AAD assay, where double negative (viable) cells were sorted using BD FACSAria cell sorter (BD Bioscience, San Jose, CA, USA) and designated as Z/100R, Z/250R, and Z/500R cells, respectively. The response curves of BTZ on parental cells and resistant cells at different degrees were plotted at 24 h and LC50 was calculated using GraphPad Prism software (La Jolla, CA, USA).

### 4.4. Hoechst 33342 Apoptosis Assay

Cells were incubated with 10 μg/mL Hoechst 33342 for 30 min and analyzed for apoptosis by scoring of cells having condensed (brighter than non-apoptotic) and/or fragmented nuclei by fluorescence microscopy (Eclipse Ti-U with NiS-Elements, Nikon, Tokyo, Japan). Faded nuclei, an indication of karyolysis, were sometimes observed. The apoptotic index was calculated as the percentage of cells with apoptotic nuclei over the total number of cells.

### 4.5. Annexin V/7-AAD Assay

Cells were harvested, washed, and stained with Annexin V-PE in binding buffer and 7-AAD for 15 min at room temperature. Samples were analyzed by BD FACSCanto flow cytometer (BD Bioscience) using a 488-nm excitation beam and 575-nm and 670-nm band-pass filters with CellQuest software. Subsequent analysis was performed by Flowjo (v10.4.1) software.

### 4.6. RNA Isolation and qPCR

Total RNA was prepared using TRIzol reagent (Invitrogen, Carlsbad, CA, USA) according to the manufacturer’s protocols, and cDNA was prepared using SuperScript III First-Strand Synthesis System and oligo (dT) primers (Invitrogen). qPCR analysis was carried out on a CFX384 Real-Time PCR (BioRad, Hercules, CA, USA) using a SYBR Green PCR Master Mix (Applied Biosystems, Waltham, MA). Initial enzyme activation was performed at 95 °C for 10 min, followed by 40 cycles of denaturation at 95 °C for 15 s and primer annealing/extension at 60 °C for 1 min. Relative expression of each gene was normalized against the housekeeping *GAPDH* gene product. Heatmap and cluster analysis were performed using MultiExperiment Viewer (MeV 4.9.0) software.

### 4.7. BioCarta Pathway Analysis

Lists of significantly changed genes comparing parental Z/Parent and BTZ-resistant Z/500R cells (*p* < 0.05) were input into DAVID (v6.8) Bioinformatics Resources, a comprehensive set of functional annotation tools. BioCarta pathways were then analyzed and visualized.

### 4.8. Overexpression Plasmid and Retroviral Transduction

Retroviral plasmid carrying human Bcl-xL transgene (3541 pMIG Bcl-XL) and GFP selection marker was a kind gift from Prof. Stanley Korsmeyer (Addgene #8790) [50]. Retrovirus production was performed using Platinum-A packaging cell lines (Cell Biolabs, San Diego, CA). Cells were incubated with Bcl-xL viral particles in the presence of hexadimethrine bromide (HBr) for 48 h and the transduced cells with GFP (FITC)-positive cells were enriched by BD FACSAria cell sorter (BD Bioscience) and analyzed prior to use by Western blotting.

### 4.9. CRISRP/Cas9-Mediated BAX Knockdown

The all-in-one lentiviral plasmid carrying *Streptococcus pyogenes* Cas9 (SpCas9), puromycin resistance gene, and sgRNA sequence specific to human *BAX* was a kind gift from Prof. Stephen Tait (Addgene #129580) [51]. The plasmid was transfected into HEK293T packaging cells in conjugation with pCMV.dR8.2 dvpr lentiviral packaging and pCMV-VSV-G envelope plasmids (Addgene #8454 and 8455) [52] to produce lentiviral particles. The particles were transduced into parental cells or Bcl-xL-overexpressed cells in the presence of 8 μg/mL HBr for 48 h and were treated with 1 μg/mL puromycin for 1 month for the selection of stably transfected cells. Depletion of Bax was confirmed by Western blotting prior to the experiments.

### 4.10. Western Blot Analysis

Cells were incubated in lysis buffer (Cell Signaling Technology) and protease inhibitor mixtures (Roche Molecular Biochemicals, Indianapolis, IN, USA) at 4 °C for 30 min. The protein concentration was determined using bicinchoninic acid (BCA) assay (Pierce Biotechnology, Rockford, IL, USA). Proteins (30–50 µg) were separated by 12% SDS-PAGE and transferred onto PVDF membranes. Membranes were blocked with 5% non-fat milk in TBST for 1 h at room temperature. Subsequently, membranes were incubated with appropriate primary antibodies overnight at 4 °C and incubated with secondary antibodies for 1 h at room temperature. Immunoblots were detected by enhanced the chemiluminescence system and visualized on an ImageQuant^TM^ digital imager (GE Healthcare, Pittsburgh, PA, USA).

### 4.11. Gene Microarray Dataset and Survival Analysis

The published dataset Gene Expression Omnibus (GEO) accession number GSE10793, which was obtained from tumor biopsies from 71 untreated MCL patients, was used to calculate overall survival associated with *BCL2A1*, *BAX* and other related genes [28]. Genechip measurements were performed using NCI/Staudt human 15K v13 arrays. Kaplan-Meier survival plot of MCL patients was generated using GraphPad Prism software according to the level of gene expression with the cutoff value for high expression at upper quartile (Q3). Expression values below Q3 were defined as low expression.

### 4.12. Statistical Analysis

The data represent means ± SD from three or more independent experiments as indicated. Statistical analysis between two groups was performed by two-sided Student’s *t*-test at a significance level of *p* < 0.05.

## 5. Conclusions

In conclusion, we provide substantial evidence that the Bcl-2 family proteins Bcl-xL and Bax are key mediators in determining BTZ sensitivity in MCL cells. Overexpression of Bcl-xL worked cooperatively with depletion of Bax to protect MCL cells against BTZ-induced apoptosis, causing acquired BTZ resistance. Co-targeting Bcl-xL and Bax, e.g., by using Bcl-xL inhibitor and Bax activator is, therefore, a promising strategy for overcoming resistance to BTZ and/or other chemotherapeutic drugs in MCL cells, in which underlying mechanisms involved the imbalance or aberrant of Bcl-2 family proteins, to improve clinical outcome and achieve long-term control of disease. It is worth noting that several selective drugs designed to inhibit antiapoptotic Bcl-2 family proteins, termed BH3 mimetics, have been developed. Our findings support recent efforts in small molecule drug discovery that Bax also serves as a promising target and that direct Bax activators could be a new class of drug candidates for cancer therapy.

## Data Availability

The data used to support the findings of this study are included within the article.

## Supplementary Materials

The following supporting information can be downloaded at: https://www.mdpi.com/article/10.3390/ijms232214474/s1.
