# Supplementary material for: Bcl-2 Family Members Bcl-xL and Bax Cooperatively Contribute to Bortezomib Resistance in Mantle Cell Lymphoma
Source: Int J Mol Sci. 2022 Nov 21;23(22):14474. doi: 10.3390/ijms232214474 (PMC9695253; doi:10.3390/ijms232214474)
Supplement: Supplementary file 1 [file ijms-23-14474-s001.zip › ijms-1989885-supplementary.pdf]

Supplementary Materials

Bcl-2 family members Bcl-xL and Bax cooperatively contribute to bortezomib resistance in mantle cell lymphoma

Sudjit Luanpitpong [1,*], Montira Janan [1,†], Juthamas Yosudjai [1,†], Jirarat Poohadsuan [1], Pithi Chanvorachote [2,3] and Surapol Issaragrisil [1,4,5]

1 Siriraj Center of Excellence for Stem Cell Research, Faculty of Medicine Siriraj Hospital, Mahidol University, Bangkok 10700, Thailand
2 Department of Pharmacology and Physiology, Faculty of Pharmaceutical Sciences, Chulalongkorn University, Bangkok 10330, Thailand
3 Cell-based Drug and Health Product Development Research Unit, Faculty of Pharmaceutical Sciences, Chulalongkorn University, Bangkok 10330, Thailand
4 Division of Hematology, Department of Medicine, Faculty of Medicine Siriraj Hospital, Mahidol University, Bangkok 10700, Thailand
5 Bangkok Hematology Center, Wattanosoth Hospital, BDMS Center of Excellence for Cancer, Bangkok 10310, Thailand
* Correspondence: sudjit.lua@mahidol.edu
† These author authors contributed equally to this work.

Table S1. STR analysis of parental Z/Parent and highly BTZ-resistant Z/500R cells used in the present study.

Locus	Samples		Reference
	Z/Parent	Z/500R	Z-138 (RRID:CVCL_B077)
Amelogenin	X,Y	X,Y	X,Y
D8S1179	8,13	8,13	8,13
D21S11	30,32.2	30,32.2	30,32.2
D7S820	8,8	8,8	8,8
CSF1PO	10,11	10,11	10,11
D3S1358	16,16	16,16	16,16
TH01	6,6	6,6	6,6
D13S317	9,12	9,12	9,12
D16S539	11,11	11,11	11,11
D2S1338	19,19	19,19	19,19
D19S433	12,14	12,14	12,14
vWA	15,18	15,18	15,18
TPOX	8,8	8,8	8,8
D18S51	14,18	14,18	14,18
D5S818	11,13	11,13	11,13
FGA	20,22	20,22	20,22

Table S2. STR analysis of Jeko-1 cells used in the present study.

Locus	Sample	Reference
	Jeko-1	Jeko-1 (RRID:CVCL_1865)
Amelogenin	X,X	X,X
D8S1179	11,14	11,14
D21S11	30,33.2	30,33.2
D7S820	10,11	10,11
CSF1PO	9,12	9,12
D3S1358	15,16	15,16
TH01	7,7	7,7
D13S317	8,9	8,9
D16S539	12,12	12,12
D2S1338	17,25	17,25
D19S433	13,13	13,13
vWA	14,14	14,14
TPOX	8,8	8,8
D18S51	13,15	13,15
D5S818	10,13	10,13
FGA	21,24	21,24
